# Interpreting Breast Cancer Survival Data by the Hazard Function: Remarkable Findings from Event Dynamics

**DOI:** 10.3390/medicina56090468

**Published:** 2020-09-12

**Authors:** Romano Demicheli, William Hrushesky, Michael Retsky, Elia Biganzoli

**Affiliations:** 1 Unit of Medical Statistics, Biometry and Bioinformatics “Giulio A. Maccacaro”, Department of Clinical Sciences and Community Health, University of Milan Campus Cascina Rosa, Fondazione IRCCS Istituto Nazionale Tumori, 20133 Milan, Italy; 2 School of Pharmacy Columbia, University of South Carolina, Columbia, SC 29201, USA; williamhrushesky@gmail.com; 3 Department of Medicine, Division of Epidemiology and Biostatistics (Cancer Epidemiology), School of Medicine Charleston, Medical University of South Carolina, Charleston, SC 29405, USA; 4 Harvard T.H. Chan School of Public Health, Boston, MA 02115, USA; michael.retsky@gmail.com

**Keywords:** breast cancer, hazard function, recurrence dynamics, tumor dormancy, tumor homeostasis, microscopic metastasis acceleration

## Abstract

The report addresses the role of the hazard function in the analysis of disease-free survival data in breast cancer. An investigation on local recurrences after mastectomy provided evidence that uninterrupted growth is inconsistent with clinical findings and that tumor dormancy could be assumed as working hypothesis to understand the clinical course of the disease. Additionally, it was deemed that the lag-time between primary tumor removal and tumor recurrence is dynamically dependent on the subclinical metastasis development within the host-tumor system and, therefore, may be informative about the biology of the disease. Accordingly, the hazard function, which estimates the event risk pattern through the time, was adopted to analyze survival data. The multipeak pattern of the hazard function suggested that the process metastasis development has discontinuous features. A new paradigm of breast cancer metastatic development was proposed, involving the notions of tumor homeostasis, tumor quiescence in specific metastatic microscopic phases and surgery-related acceleration of the metastatic process. All analyses by prognostic factors (e.g., by menopausal status) or treatment modalities (e.g., by adjuvant chemotherapy) or other parameters (e.g., site of metastasis), provided coherent data in agreement with the model. The hazard rate function allowed addressing several clinical questions including meaning of ipsilateral breast tumor recurrence (IBTR), oncologic effect of delayed breast reconstruction, surgery related metastasis acceleration, possible role of anti-inflammatory drugs and body mass index (BMI) to modulate the recurrence risk. We conclude that the hazard function is a powerful tool to investigate the post-surgical course of early breast cancer and other operable tumors and to make inferences on their biology.

## 1. Tumor Dormancy and the Hazard Function: A Joint History

In breast cancer, metastases or local recurrence appear at variable time intervals (from a few months to many years) after primary treatment. Two conflicting hypotheses were being considered in the mid-1990s to explain this phenomenon: The first one was founded on uninterrupted tumor growth [1], while the second one assumed some type of growth control maintaining the tumor in a “dormant” state [2]. Nevertheless, although both hypotheses were supported by many arguments, each lacked convincing experimental data. The question was resolved for local recurrences after mastectomy by the analysis we carried out on the time to recurrence in 122 patients who underwent periodical follow-up examinations [3]. This investigation provided evidence that the hypothesis of uninterrupted growth involves a statistically significant departure from observed data and, therefore, should be rejected. The results, moreover, suggested that a time span of tumor dormancy followed by quite rapid growth could provide a more reasonable description of the observed recurrence pattern. Following this main conceptual change of the understanding of local recurrences, the question emerged whether tumor dormancy was able to explain the metastatic course for distant recurrences as well. 

This question forced us to reconsider the techniques that are usually adopted to analyze clinical events occurring during the follow-up. In clinical trials, the time-to-event variables were (and still are) near universally described by survival curves estimating the probability that a patient has not experienced the event under investigation within a given interval following a suitable time origin. For instance, in breast cancer, the recurrence-free survival curves provide the percentage of patients who remain recurrence-free at a given time after primary tumor removal. This approach focuses on the cumulative event-free time distribution, i.e., cumulative incidence risk, and affords poor information about the recurrence dynamics, i.e., the timing of tumor recurrence, which can be of considerable interest. Indeed, the lag-time between primary tumor removal and tumor recurrence is related to the state of microscopic metastatic foci at the time of surgery and to their growth pattern during the subclinical phase. Therefore, the recurrence dynamics during the follow-up could provide insights on the biological behavior of metastases. The recurrence dynamics specifically may be suitably described by the hazard function, i.e., the instantaneous event rate throughout the follow-up time, which, in a discrete framework, is represented by the conditional event probability at different time intervals, given survival before that time. The analysis of disease-free survival data by the hazard function had been seldom utilized in the past, e.g., [4], and even a statistical paper published in a very popular oncologic journal [5] did not result in improved frequency of use. 

In the light of this reasoning, an investigation on the time distribution of treatment failures was carried out [6] employing the cause-specific hazard function for loco-regional and/or distant recurrence. Recurrence as first event was considered the outcome of interest, and other events that either may hinder it or modify the chance that it occurs, for instance, non-cancer related events, were considered “competing events”. Accordingly, the estimate of risk of recurrence versus time was assessed by techniques taking into account competing risks. The results of this study established that the hazard rate for recurrence shows a multipeak pattern, which implies that the main process of overt clinical metastases appearance has discontinuous features, inconsistent with uninterrupted tumor growth since tumor seeding. On the contrary, the tumor dormancy concept immediately appeared able to easily explain the new findings. Indeed, one could assume that at the time of primary tumor removal, microscopic metastatic foci may be in different biological steady states, most of which are dormancy states and that they start growing at the time of primary tumor surgical removal, thereafter, producing the observed clustered hazard pattern.

## 2. A New Paradigm for the Metastatic Development

These findings, as well as associated computerized simulations, encouraged a new paradigm of breast cancer metastatic development [7,8], involving the concepts of tumor homeostasis, tumor quiescence in specific metastatic microscopic phases (single cells and not vascularized foci) and surgery-related acceleration of the metastatic process. According to the model, metastatic foci existing at the time of primary tumor removal were assumed to stay in two dormant states: (1) single cells or nests containing a few cells, where most of them are non-dividing [9] and (2) non-vascularized micro-metastases with size less than 1–2 mm [10]. Orderly transitions between these dormant states eventually originate the appearance of clinical metastases. Primary tumor, however, could exert homeostatic effects upon its metastases and concur to, if not determine, the metastatic dormancy [11]. Therefore, primary tumor surgical removal may abolish homeostatic restraints allowing abrupt acceleration of the metastatic process owing to single tumor cell proliferation and/or vascular access of micrometastases [12,13]. This should be considered an undesirable effect of surgery, which occurs together with the favorable effect of achieving a long-lasting disease-free state in a significant percentage of patients. We must realize that the host–tumor–treatment biology may imply additional effects to be addressed in order to improve the effectiveness of their therapeutic action. We are still far from knowing well the phenomena involved in the standard treatment of the disease, which, it must be remembered, can be target of newly considered therapies. Recently, the effect of primary tumor surgical removal, which was at that time hypothesized, has been directly evidenced for tumor cell deposits whose outgrowth was restricted by the adaptive immune system [14,15]. 

The analysis of recurrence dynamics was then adopted to investigate subsets of patients displaying specific characteristics, with the underlying purpose of testing the proposed model. Indeed, as it is true for all scientific hypotheses and theories, the validity of the model cannot be demonstrated but only falsified, namely, it should resist in the face of the challenge of explaining further and further clinical data. Among others, a few main supportive steps were resolved when the recurrence dynamics was analyzed by menopausal status and by adjuvant chemotherapy (yes vs. not). 

The recurrence dynamics by premenopausal status for patients undergoing mastectomy as unique treatment provided evidence that the mortality increase associated with larger tumors (>2 cm vs. <2 cm) is correlated to the menopausal status at cancer diagnosis and on the follow-up time [16]. During early follow-up, the different mortality dynamics associated to menopausal status show that the reduction of death rate from tumor downsizing is less important for premenopausal than for postmenopausal women. This finding is relevant to the early mortality paradoxical excess in the invited group, for women age 40 to 49 years, that was observed in all mammography screening studies [17]. Indeed, mammography screening provides earlier diagnosis in the invited group in comparison with the control arm, resulting in earlier primary tumor removal and, according to the model, in earlier surgery-induced recurrence and death. This harmful trend counteracts the mortality reduction due to earlier diagnosis, which, unfortunately, is lower in premenopausal than in postmenopausal women, at least during the first few years following surgery. Therefore, this event dynamic would display favorable balance only in the postmenopausal group. 

**Figure 1:**
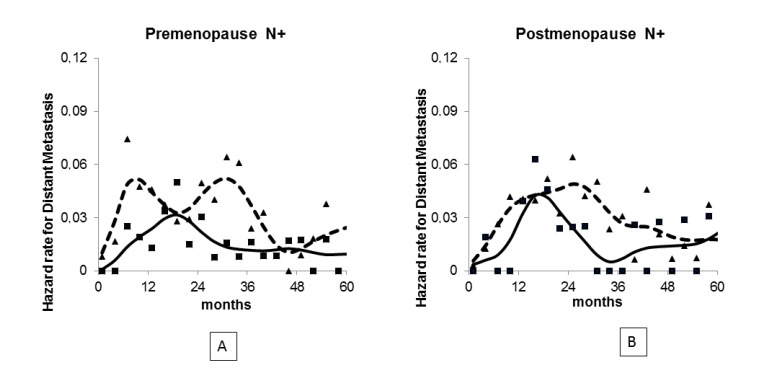
Hazard rate for distant recurrence in premenopausal (**A**) and postmenopausal (**B**) patients given no adjuvant systemic treatment (dashed line) or adjuvant Cyclophosphamide, Methotrexate and Fluorouracil for six courses (continuous line). Both point estimates (on a three-month time lag) and smoothed curves are reported.

About menopausal status, a detailed analysis of the first peak of the distant metastasis (DM) dynamics [18] revealed a two-peaked hazard function for premenopausal patients (Figure 1A, red curve), whereas the corresponding growth pattern in postmenopausal patients displayed a wider single peak (Figure 1B, red curve) without evident substructure, thus disclosing menopausal-status related features in keeping with findings about the age distribution of prognostic factors. When this recurrence pattern for untreated patients was compared with the corresponding pattern of patients receiving adjuvant chemotherapy with Cyclophosphamide, Methotrexate and Fluorouracil (CMF) [19], very instructive results emerged. Indeed, the analysis displayed that the CMF-related hazard reduction occurs during the first four years after primary tumor surgical removal and is mainly restricted to two temporally separate recurrence clusters during the first and third years of follow-up, while the second-year recurrences are weakly affected (Figure 1). Notably, these effects upon the recurrence dynamics are menopausal status-independent in such a way that the hazard rate curve for patients given adjuvant chemotherapy assumes the same pattern in pre- and postmenopausal women (Figure 1, blue curves). These findings strongly suggest that at least two different therapeutically vulnerable proliferative events occur during the administration of adjuvant chemotherapy and that post-resection mechanisms by which chemotherapy prevents metastases are similar, but of different magnitude in pre- and postmenopausal women. Ultimately, they are consistent with the proposed metastasis model. 

## 3. The Hazard Function Clarifies Clinical Questions

The analysis approach by means of the hazard function allowed addressing several additional clinical questions including the meaning of ipsilateral breast tumor recurrence (IBTR), delayed breast reconstruction, surgery related metastasis acceleration, possible role of perioperative anti-inflammatory drugs and body mass index (BMI) to modulate the recurrence risk. In the following, these subjects will be briefly addressed.

The significance to be attributed to IBTR in breast cancer patients undergoing breast conserving treatment has been a highly controversial issue since the early reports. According to some researchers [20], IBTR after BCT does not change the course of the disease and is considered an important marker of increased risk, not a cause, of DM. Conversely, others [21] view IBTR as a source of new DM causing increased subsequent mortality. We carried out the analysis of the hazard rate for further recurrence and mortality in a timeframe with time-origin at the surgical treatment for IBTR [22,23]. We found a bimodal pattern matching to the corresponding dynamics following primary tumor removal in node positive (N+) patients. These findings unquestionably would support Fisher’s concept [20] that IBTR is a marker of intrinsic high risk of DM, which was undetectable at the initial treatment and is revealed by the IBTR emerging in advance of the competing DM event. Moreover, the clinical course may be properly explained by observing that the surgical maneuver required by IBTR treatment may activate a sudden growing phase for tumor foci, most of which would have reached the clinical level according to their own dynamics. As a corollary, the sudden increase of the recurrence risk at the IBTR surgical removal suggests the occurrence of metastatic growing phases sensitive to cytotoxic treatments. Therefore, treating patients in this dynamic phase would be effective, like the first adjuvant systemic treatment.

The safety of delayed reconstructive surgery has been questioned. Indeed, local and systemic growth signaling cascades originated from tissue trauma, and wound healing could alter the dormant state of occult micrometastases [24]. In breast cancer, tissue trauma due to primary surgery has been associated with tumor progression [25] as well. Increased as well as reduced risk of recurrence were observed after delayed breast reconstruction, fostering a debate among plastic surgeons [26,27]. The question became more readable when a suitable database of patients undergoing delayed reconstruction after mastectomy was analyzed [28] by placing the time origin at the reconstruction maneuver or adopting the multiple time scale analysis. It was revealed that there is a transitory significant increase of recurrence risk lasting about 2 years, thus providing evidence that the reconstructive surgery may exert an enhancing effect on subclinical metastases. Moreover, the analysis suggested that this peak is caused by metastatic events that, missing reconstruction, were to be expected later. In other words, although reconstructed patients display increased recurrence rate, the long-term disease-free survival is not worse than that of not reconstructed patients.

Main details of the surgery related acceleration of metastasis development could be revealed by comparing DM dynamics following subsequent surgeries after primary tumor removal, because of IBTR, contralateral breast cancer or delayed breast reconstruction [29]. We could ascertain that the impact of breast tumor surgical removal on microscopic metastases apparently involves two different factors, i.e., tumor homeostasis interruption and surgical wound effects. The sudden elimination of the restrains of primary tumor on metastatic foci would allow metastasis development, which, in turn, would be supported by the promoting mechanisms triggered by the surgical wounding. 

A retrospective study [30] comparing various perioperative analgesics and anesthetics during mastectomy for early breast cancer suggested that ketorolac, a NSAID used in surgery for analgesia, is associated with better disease-free survival. 

Despite the short follow-up, the hazard rate pattern for recurrence of patients receiving the drug displayed a definite reduction of the expected early peak [31]. The report should be cautiously considered and needs confirmation by a well-designed randomized clinical trial. If confirmed, the finding of using a widely tested, safe and cheap anti-inflammatory agent to reduce early metastases has obvious prominent consequences mainly in low income countries. A plausible rationale, supporting the possible activity of ketorolac, is from the well documented surgery-related systemic inflammation, which might be an enhancing factor of the metastatic process and could be blocked by perioperative NSAIDs. 

A position of inflammation in shaping the course of breast cancer is even suggested by the role that adiposity, most likely via the associated chronic low-grade inflammation, plays in modulating the recurrence risk. In a recent report, among node-positive breast cancer patients receiving adjuvant chemotherapy regimens, subsets based on estrogen receptor (ER) status, menopausal status and BMI value (normal weight, overweight and obese) were analyzed [32]. Overweight and obese patients mainly account for the hazard of late relapses in ER-negative patients. Conversely, in premenopausal patients with ER-positive tumors, obesity was associated with both early and late peaks of DM, while overweight patients displayed intermediate risks between obese and normal-weight patients. In postmenopausal patients with ER-positive tumors, the distant recurrence rate was significantly higher in the overweight patients compared to the other BMI categories, and obese patients displayed a second late recurrence peak as well. These results show that recurrence dynamics is associated with patient’s BMI at diagnosis. This interaction was further observed in a large database when the effect of perioperative ketorolac or diclofenac on distant metastasis incidence was analyzed [33]. The time-dependent analyses showed that ketorolac administration was especially associated with early recurrence reduction in patients with increased BMI. This association is apparently more noticeable for overweight patients older than 50 years of age. The administration of diclofenac did not display any benefit, thus suggesting that subtle differences between otherwise similar drugs may be important. 

## 4. How to Achieve the Best from the Hazard Function?

Findings observed for breast cancer were confirmed in different databases (e.g., [34,35]) and, beyond breast cancer, the analysis of recurrences by the hazard function was adopted in other tumors, including non-small cell lung cancer [36,37], ocular melanoma [38], and gastric cancer [39] where multi-peak curves were detected, suggesting that the reported dormancy based model, far from being limited to breast cancer, may have wide applicability. However, in spite of this general agreement among findings in breast cancer reports, a few researchers were not able to reproduce the same results (e.g., [40,41]). This occurrence needs closer examination. 

The first point to be considered is proper determination of the time to event. Beyond adequate patient number, quality of data is a basic requisite for uncovering the shape of hazard rate curve. This quite-obvious point is not especially critical when the analysis is aimed at estimating the cumulative event-free time, namely, the percentage of patients who remain recurrence-free at a given time (usually after a number of years of follow-up). On the contrary, the hazard rate estimates need high accuracy in the time to event assessment, i.e., in follow-up data collection. The hazard function is like a variable-over-time signal, which provides information, the readability of which is affected by the background noise as it occurs for all signals. Therefore, although the usual accuracy of time data collecting is quite sufficient for standard evaluation of clinical cumulative effects, it may be (and often is) suboptimal for event dynamics investigations.

**Figure 2:**
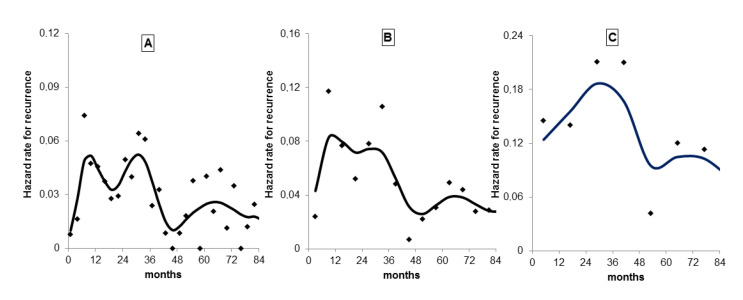
Hazard rate for distant recurrence in premenopausal patients undergoing mastectomy only. Distant recurrences were analyzed adopting different time windows. Events in a 3-month time lag (**A**) allowed detecting the fine structure that is poorly observable in the 6-month analysis (**B**) and is undetectable in a 12-month analysis (**C**). Point estimates, standard deviations and smoothed curves are reported. The effects of the different bias-variance tradeoffs are quite evident.

A second point regards the discretization of time, which is crucial for detecting hazard rate structures. Several reports adopted annual rates of recurrence, a choice that allows more stable estimates but prevents the identification of relevant time patterns. In homogeneous case series with accurate follow-up collection, a continuous analysis of time according to a finer discretization is crucial for detecting hazard rate structures (according to the bias-variance trade-off principle of non-parametric estimation). Analyzing events in a 3-month time lag allowed detecting the fine structure of premenopausal recurrence risk during the first years of follow-up [18] that was elusive in the previous 6-month analysis [6] and that is undetectable in a 12-month analysis (Figure 2). 

A third point involves the definition of the analyzed variable, which should be unequivocal and clinically relevant (e.g., distant metastasis, death for any cause, etc.) In this regard, it is enlightening to examine a relevant paper focused on the limit of breast cancer dormancy [41], where the authors “did not see evidence for a second peak in the hazard curve”. This fact may be attributed to the event they analyzed (“first recurrence or death from breast cancer”). Indeed, while “first recurrence” and “death from breast cancer” possibly identify treatment failure, the combined event is likely to be not appropriate for studying the time-dependent structure of the recurrence dynamics. A clue supporting this interpretation is the occurrence that, when comparing the yearly discrete hazard for time to death from all causes from reports [6] and [41], both curves displayed impressive similarity in patterns [42].

## 5. Conclusions

This short review, covering more than twenty years of investigations on breast cancer, suggests a few thoughts that are extendable to other malignancies as well. 

Time is a fundamental factor in the cancer course and should be extensively investigated at all levels, including the clinical one, to understand disease development modalities and hence therapy opportunities. Although it seems an obvious thought, we can see that accepting this concept and putting it into practice is difficult, especially in clinical investigations. Currently, several clinical trials are still focused on a simplistic question about treatment efficacy (effective vs. not/less effective), looking at cumulative probability endpoints and hence disregarding valuable information enclosed in the changes of disease course dynamics provided by the hazard function estimation instead. 

All treatment modalities have side effects, a few of which may be detrimental and, if ascertained, need specific management. The accelerating effect of breast surgery on the development of microscopic dormant metastases is a typical example. Nevertheless, breast surgeons should not feel guilty (as we have sometimes observed): This phenomenon does not invalidate the established beneficial influence of primary tumor resection on the natural history of the disease. Nevertheless, the knowledge of dormancy and dormancy interruption mechanisms (e.g., inflammatory consequences of surgery) may open new investigational areas (e.g., the employment of perioperative anti-inflammatory drugs) that will enhance the role of primary tumor surgical removal, while reducing its detrimental side effects. 

Adopting the hazard rate function to analyze recurrence patterns allowed us to identify clinical behaviors supporting unanticipated aspects of tumor biology, such as tumor homeostasis. While “cancer cells” still occupy the center of the stage in several oncologic investigations, perpetuating a reductionist approach, clinical findings support the concept of “cancer tissue” and hence of tumor as an “organ-like structure”, according to a systemic approach, which is progressively emerging even from laboratory investigations [43]. It is instructive to acknowledge how a slightly different way to look at clinical data by an appropriate analytical tool was capable to open a fruitful landscape for better understanding breast cancer. 

Finally, a comment on present cancer research trends. The idea that cancer is an exclusively genetic disease has in the last decades “sucked all the air out of the room”. Its dominance has overwhelmed imaginative cancer research. It has diminished creative thought, which may sometimes much better explain relevant cancer biology. This is true for almost all cancers and very true for breast cancer. The above outlined large effects of menopausal state at the time of primary tumor resection upon the subsequent recurrence and survival dynamics illustrate the relevance of host milieu to breast cancer biology and even survival many years hence. Let us take as another example the fact that half the world’s breast cancers occur and are treated among cycling pre-menopausal women, whose hormonal milieu is changing greatly each month. More than a dozen clinical trials have suggested superior outcome among women whose cancers are resected during the early luteal phase of the cycle compared with women whose cancers are resected during the mid and late luteal phase [44,45]. Genetics are not irrelevant, as breast cancer gene expression is likewise modulated during each menstrual cycle in ways that shed light upon the causes of predictable menstrual cycle dependent breast cancer recurrence and survival dynamics [46]. It remains clear that genetic mutations within breast cancer cells affect their biology. It remains equally clear that resonating host dynamics predictably modulate cancer growth and spread and the survival of each patient within whom these cancers reside.
